# Prognosis and influencing factors of follicular thyroid cancer

**DOI:** 10.1002/cam4.6727

**Published:** 2023-12-16

**Authors:** Jiafei Shen, Meiying Yan, Long Chen, Di Ou, Jincao Yao, Na Feng, Xueqin Zhou, Zhikai Lei, Dong Xu

**Affiliations:** ^1^ Zhejiang Cancer Hospital, Hangzhou Institute of Medicine (HIM), Chinese Academy of Sciences Hangzhou China; ^2^ Zhejiang University School of Medicine, Affiliated Hangzhou First People's Hospital Hangzhou China; ^3^ Esaote SpA Genova Italy

**Keywords:** follicular thyroid cancer, metastasis, prognosis

## Abstract

**Objectives:**

Follicular thyroid cancer (FTC) is prone to distant metastasis, and patients with distant metastasis often have poor prognosis. In this study, the impact of metastasis and other relevant factors on the prognosis of follicular thyroid carcinoma was examined.

**Methods:**

This was a retrospective study. Data were obtained from Zhejiang Cancer Hospital, Sun Yat‐sen University Cancer Center and Hangzhou First People's Hospital affiliated with Zhejiang University School of Medicine, from January 2009 to June 2021 for 153 FTC patients. The patients were assigned into three groups according to their distant metastasis: distant metastasis at initial diagnosis (M1), distant metastasis during follow‐up (M2), and no evidence of distant metastasis over the course of the study (M0). Data were collected and summarized on clinical data, laboratory parameters, imaging features, postoperative pathologic subtypes, and metastases. The Cox proportional hazard model was used to perform the univariate and multivariate analysis. Kaplan–Meier curves were used to evaluate cancer‐specific survival (CSS).

**Results:**

Based on metastasis, the patients were assigned into three groups, including 31 in the M1 group, 15 in the M2 group, and 107 in the M0 group. These individuals were followed up for an average of 5.9 years, and the group included 46 patients with distant metastasis (31 confirmed at diagnosis and 15 found during follow‐up). Univariate Cox regression analysis showed that age, Hashimoto's thyroiditis (HT), surgery method, postoperative adjuvant therapy, histologic subtype, nodule size, calcification, TSH, and distant metastasis all impacted prognosis. Multivariate Cox regression analysis suggested that histologic subtype (widely invasive; HR: 7.440; 95% CI: 3.083, 17.954; *p* < 0.001), nodule size (≥40 mm; HR: 8.622; 95% CI: 3.181, 23.369; *p* < 0.001) and distant metastasis (positive; HR: 6.727; 95% CI: 2.488, 18.186; *p* < 0.001) were independent risk factors affecting the prognosis of follicular thyroid cancer.

**Conclusions:**

Histologic subtype, nodule size, and distant metastasis are important risk factors for the prognosis of follicular thyroid cancer. Patients with metastatic follicular thyroid cancer have a poor prognosis, especially with metastasis at the time of initial diagnosis. As a result, this group of patients requires individualized treatment and closer follow‐up.

## 1 INTRODUCTION

**FIGURE 1:**
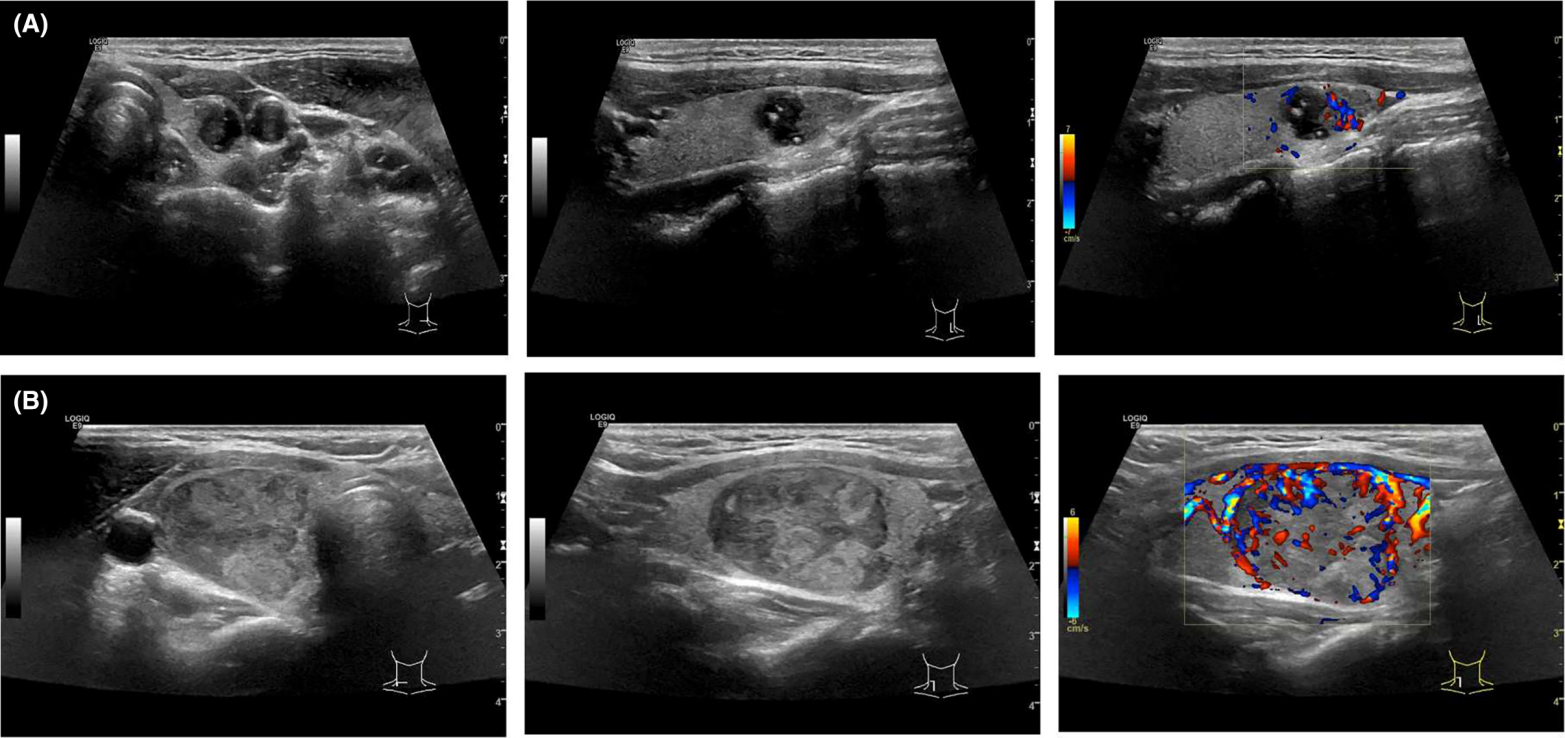
(A) Ultrasound images of PTC. (B) Ultrasound images of FTC.

Thyroid cancer is the most common endocrine malignancy, with an increasing incidence worldwide.^1^ Follicular thyroid cancer (FTC) is the second most common malignancy originating from follicular thyroid cells, accounting for about 10%–15% of all thyroid cancer.^2^, ^3^ There have been many previous studies on the diagnosis, treatment, and prognostic factors of papillary thyroid carcinoma (PTC), but relatively few studies on FTC. FTCs are generally difficult to diagnose in preoperative cytology and imaging procedures, but usually confirmed in postoperative histopathologic examination.^4^, ^5^ A follicular neoplasm (Bethesda IV) found in fine‐needle aspiration biopsy (FNAB) had a risk of malignancy at 10%–40%, compared to 6%–28% for atypia of undetermined significance/follicular lesion of undetermined significance (Bethesda III).^6^ However, the national malignancy rate is higher than these rates in fact.^7^ The comparison of the ultrasonography of PTC and FTC in some studies has found that FTC generally has a larger tumor size and tends to be isoechoic, it also usually lacks the suspicious sonographic features found in PTC, such as aspect ratio >1 and microcalcification^8^ (Figure 1).

Lymph node metastases are uncommon in patients with FTC, but metastases to distant organs are common, such as the lungs and bones.^2^ Distant metastasis to bone is often found at diagnosis, while distant metastasis to lungs is often found after surgery. Some studies have reported that the prognosis of patients with bone metastasis is usually worse than that of patients with lung metastasis.^9^ Distant metastasis is considered the most important prognostic factor for FTC survival.^10^, ^11^ Distant metastasis may be the initial manifestation of FTC. Age and extrathyroidal invasion have been reported to be significant risk factors for FTC distant metastases.^12^, ^13^, ^14^ FTC patients with distant metastasis, especially when it is found at diagnosis, have poor survival outcomes. Therefore, compared to PTC, FTC is more aggressive and has a worse prognosis,^15^ regardless of whether distant metastasis is present at diagnosis or not, and regardless of whether any metastasis is present after initial thyroid surgery.^2^ It has also been suggested that nodule size and histologic subtype are factors influencing prognosis, such that patients with nodules >4 cm and widely invasive FTC have a worse prognosis. Studies on the influence of surgical method on the prognosis of FTC patients do not have the same results, some believe that the prognosis of unilateral thyroidectomy is similar to that of total lobectomy, while others prefer a better prognosis for total lobectomy.^3^, ^16^, ^17^, ^18^, ^19^, ^20^ Therefore, the choice of surgical method by surgeons is still controversial.

The studies related to the prognosis of FTC include more scattered factors and the results are not entirely consistent. In view of these circumstances, it is necessary to go to an in‐depth study to summarize the prognosis of FTC patients and its associated factors. In this study, we retrospectively collected and analyzed the prognosis of FTC and the risk factors that may affect the prognosis, including nodule size, histologic subtype, surgical method, in order to guide the clinical management of FTC patients and their later follow‐up.

## 2 MATERIALS AND METHODS

### 2.1 Data source

The present study was a retrospective study. In this study, the information of patients treated for follicular thyroid cancer in recent years at Zhejiang Cancer Hospital, Sun Yat‐sen University Cancer Center, Hangzhou First People's Hospital was collected retrospectively. The clinical characteristics, laboratory data, imaging results, pathologic results, and follow‐up information for each patient were recorded. The follow‐up was mainly carried out through the electronic medical record follow‐up system, partially by telephone.

### 2.2 Screening and inclusion of covariates

There were 199 FTC patients who visited the hospital between January 2009 and June 2021. Pathologic diagnosis and categorization of follicular carcinoma based on the latest WHO classification system. The inclusion criteria were: patients with single thyroid lesion, surgery at the three previously mentioned hospitals, surgical pathology confirmed as follicular carcinoma, regular postoperative follow‐up and review, complete and non‐deficient clinical data, and no local recurrence. The exclusion criteria were: preliminary surgery at other facilities, lost to follow‐up, missing medical data, multiple thyroid lesions, and local recurrence. Single thyroid lesion or not based on pathology. The final 153 patients were included in further analysis and divided into three groups based on their distant metastases: 31 patients with distant metastases at initial diagnosis (M1), 15 patients with distant metastases during follow‐up (M2), and 107 patients with no evidence of distant metastases during the study (M0). We identified and included common variants that might influence prognosis. These included aspects of basic clinical data, laboratory parameters, imaging features, postoperative pathologic subtypes, and metastasis. The covariates identified were age at initial surgery (the age cutoff was set at 55 years based on ATA guidelines and the current (8th edition) AJCC/TNM staging system), gender, thyroid‐stimulating hormone (TSH; categorized as normal, increasing, decreasing according to normal range criteria 0.38–4.340 μIU/mL), Hashimoto's thyroiditis (HT; positive for either anti‐thyroid peroxidase antibody or anti‐thyroglobulin antibody, otherwise negative), ultrasound signs (read by an experienced ultrasonographer, including halo, calcification, and tumor aspect ratio), nodule size, surgery method, postoperative adjuvant therapy, and histologic subtype (minimally invasive follicular thyroid cancer [MIFTC]; widely invasive follicular thyroid cancer [WIFTC]) and distant metastasis (such as bone, lung, brain).

### 2.3 Data statistical method

The continuous variables in normal distribution were expressed as the standard deviation of the mean. Continuous variables with no normal distribution are expressed in the mean and quartile ranges (IQR). Class variables are expressed in numbers (*n*) and percentages (%). For the continuous variables in normal distribution, comparisons of features were made through calculations using independent sample *t*‐test or one‐way analysis of variance (ANOVA). Mann–Whitney *U* test and Kruskal–Wallis ANOVA were used for the continuous variables without normal distribution. Chi‐square test, adjusted chi‐square test, and Fisher's exact test were used for the categorical variables.

Cancer‐specific mortality (CSM) and cancer‐specific survival (CSS) were calculated by classification of cancer characteristics to assess the impact of classification criteria on mortality and survival rate. Univariate and multivariate COX regression analyses were performed to identify factors associated with CSM. Kaplan–Meier curves were used to evaluate CSS. Analysis was performed using SPSS 26.0; *p* < 0.05 was taken as the threshold for statistical significance.

### 2.4 Ethical approval

This study was approved by the ethics committees of all participating hospitals and all patients‐identifying information was removed. Cancer Hospital Affiliated to the University of Chinese Academy of Sciences (IRB‐2020‐287) Sun Yat‐sen University Cancer Center (SL‐B2021‐021‐02) Zhejiang University School of Medicine Affiliated Hangzhou First People's Hospital (IRB‐010‐01). Written informed consent was waived due to the retrospective nature of the study.

## 3 RESULTS

### 3.1 Flowchart of cohort establishment

**FIGURE 2:**
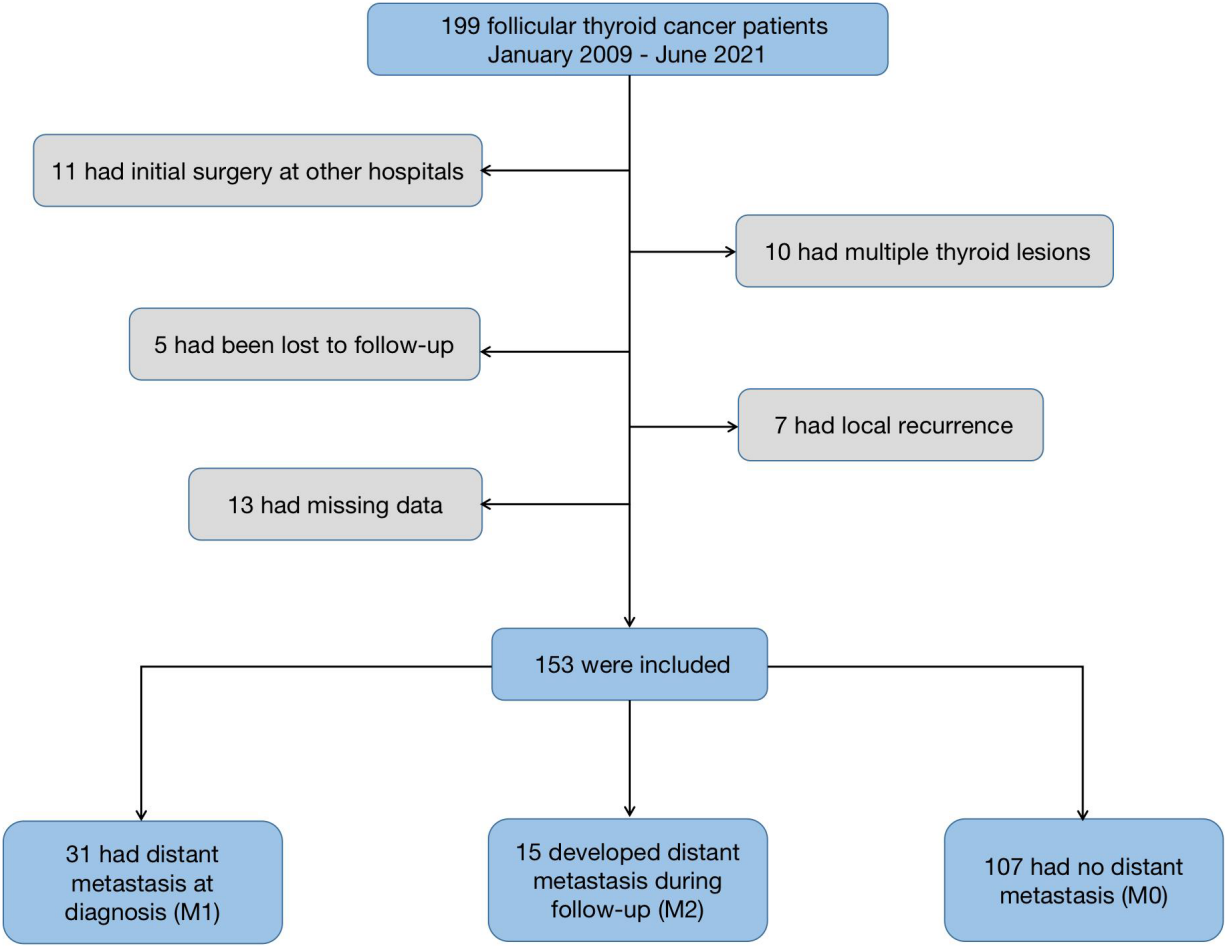
Flowchart of cohort establishment.

A total of 199 FTC patients diagnosed between January 2009 and June 2021 were screened out according to the pathologic results. Some patients were excluded, including 11 with outpatient surgery, 5 with failed follow‐up, 13 with missing medical data, 10 with multiple lesions, and 7 with local recurrence. Finally, 153 patients diagnosed with FTC between January 2009 and June 2021 were included for further analysis. Based on metastasis, the patients were assigned into three groups, including 31 in the M1 group, 15 in the M2 group, and 107 in the M0 group (Figure 2).

### 3.2 Basic clinical characteristics

The M1 group exhibited the highest average age (60.0 ± 10.7 years), followed by the M2 group (56.5 ± 16.9 years) and the M0 group (46.6 ± 14.7 years) (*p* < 0.001), with significant differences between all pairs of groups (*p* < 0.025). Gender, TSH levels, and tumor aspect ratio did not show statistically significant differences among the three groups (*p* > 0.05). HT had the highest prevalence in the M1 group (45.2%), followed by the M2 group (26.7%), and then the M0 group (21.5%) (*p* = 0.003). The Department of Pathology at the hospital classified FTC tissues into MIFTC and WIFTC. In this study, the M1 group had the highest proportion of WIFTC (67.7%), followed by the M2 group (40%), and then the M0 group (7.5%) (*p* < 0.001). Statistically significant differences were observed between any two groups (*p* < 0.001). The M1 group had the highest proportion of tumors with a size of ≥40 mm (67.7%), while the M1 group had the highest proportion of tumors with a size of <40 mm (69.2%). These differences were statistically significant both among the three groups (*p* < 0.001) and between the two groups (*p* < 0.025). Surgical method, postoperative adjuvant therapy, and calcification were all statistically significant among the three groups (*p* < 0.001). In groups M1 and M2 compared to group M0, HT, surgical method, and postoperative adjuvant therapy were statistically significant, while they were not statistically significant in groups M2 and M0 (*p* < 0.025). The M1 group had the highest CSM rate (100%), followed by the M2 group (40.0%), while the M0 group had no CSM (*p* < 0.001). The average follow‐up duration in the M1, M2, and M0 groups was 30, 69, and 68 months, respectively (*p* < 0.001) (Tables 1 and 2).

**TABLE 1:** Clinical features of 153 patients with follicular thyroid cancer.

	M1 group, *n* = 31	M2 group, *n* = 15	M0 group, *n* = 107	*p*‐value
Age (years)	60.0 ± 10.7	56.5 ± 16.9	46.6 ± 14.7	
<55	6 (19.4%)	6 (40.0%)	76 (71.0%)	
≥55	25 (80.6%)	9 (60.0%)	31 (29.0%)	<0.001
Gender, *n* (%)				
Female	17 (54.8%)	9 (60.0%)	73 (68.2%)	
Male	14 (45.2%)	6 (40.0%)	34 (31.8%)	0.427
Hashimoto's thyroiditis, *n* (%)				
No	17 (54.8%)	11 (73.3%)	84 (78.5%)	
Yes	14 (45.2%)	4 (26.7%)	23 (21.5%)	0.003
Surgery method, *n* (%)				
Unilateral lobectomy	1 (3.2%)	6 (40.0%)	54 (50.5%)	
Total unilateral lobectomy	28 (90.3%)	7 (46.7%)	31 (29.0%)	
Near‐total unilateral lobectomy	2 (6.5%)	2 (13.3%)	22 (20.5%)	<0.001
Postoperative adjuvant therapy, *n* (%)				
No	8 (25.8%)	6 (40.0%)	91 (85.0%)	
Radioiodine therapy	23 (74.2%)	9 (60.0%)	16 (15%)	<0.001
Histologic subtype, *n* (%)				
Minimally invasive	10 (32.3%)	9 (60.0%)	99 (92.5%)	
Widely invasive	21 (67.7%)	6 (40.0%)	8 (7.5%)	<0.001
Nodule size, *n* (%)				
< 40 mm	10 (32.3%)	9 (60.0%)	74 (69.2%)	
≥40 mm	21 (67.7%)	6 (40.0%)	33 (30.8%)	<0.001
Calcification, *n* (%)				
No	9 (29.1%)	7 (46.7%)	62 (57.9%)	
Bulky	5 (16.1%)	0 (0%)	10 (9.4%)	
Rim	4 (12.9%)	2 (13.3%)	5 (4.7%)	
Punctate	13 (41.9%)	6 (40.0%)	30 (28.0%)	0.002
Acoustic halo, *n* (%)				
No	30 (96.8%)	15 (100%)	83 (77.6%)	
Yes	1 (3.2%)	0 (0%)	24 (22.4%)	0.005
Tumor aspect ratio, *n* (%)				
<1	28 (90.3%)	15 (100%)	100 (93.5%)	
>1	3 (9.7%)	0 (0%)	7 (6.5%)	0.452
TSH, μIU/mL (M[IQR])	1.2 (0.6, 2.3)	1.9 (1.2, 2.6)	1.6 (1.1, 2.3)	
Normal, *n* (%)	11 (35.5%)	9 (60.0%)	83 (77.6%)	
Down	19 (61.3%)	4 (26.7%)	22 (20.6%)	
Up	1 (3.2%)	2 (13.3%)	2 (1.8%)	0.182
Cancer‐specific mortality, *n* (%)	31 (100%)	6 (40.0%)	0 (0%)	<0.001
Follow‐up, months	30.0 (4.0, 64.0)	69.0 (38.0, 125.0)	68.0 (49.0, 120.0)	<0.001

**TABLE 2:** Comparison of clinical characteristics associated with prognosis between the two groups.

	M1 group, *n* = 31			M2 group, *n* = 15		
	*n*	*p*	χ^2^	*n*	*p*	χ^2^
Age (years)		*<0.001*	*21.388*		*0.006*	*NA*
<55	*6*			*6*		
≥55	*25*			*9*		
Hashimoto's thyroiditis		*0.010*	*6.860*		*0.122*	*NA*
No	*17*			*11*		
Yes	*14*			*4*		
Surgery method		*<0.001*	*39.131*		*0.658*	*1.190*
Unilateral lobectomy	*1*			*6*		
Total unilateral lobectomy	*28*			*7*		
Near‐total unilateral lobectomy	*2*			*2*		
Postoperative adjuvant therapy		*<0.001*	*43.957*		*0.036*	*9.939*
No	*8*			*6*		
Radioiodine therapy	*23*			*9*		
Histologic subtype		*<0.001*	*52.594*		*<0.001*	*NA*
Minimally invasive	*10*			*9*		
Widely invasive	*21*			*6*		
Nodule size		*<0.001*	*13.741*		*0.001*	*NA*
<40 mm	*10*			*9*		
≥40 mm	*21*			*6*		
Calcification		*0.028*	*8.909*		*0.020*	*8.348*
No	*9*			*7*		
Bulky	*5*			*0*		
Rim	*4*			*2*		
Punctate	*13*			*6*		
^a^M0 group, *n* = 107						

^a^ The control group.

*Note*: NA This is a Fisher's exact test with no chi‐square value.

### 3.3 Risk factors associated for cancer‐specific mortality

Univariate Cox regression analysis showed that CSM was significantly associated with the following factors: age (≥ 55 years; HR: 8.636;95% CI: 3.535, 21.097; *p* < 0.001), HT (HR: 2.372; 95% CI: 1.242, 4.530; *p* = 0.009), surgery method (total or near‐total unilateral lobectomy; HR: 0.339;95% CI: 0.188, 0.611; *p* < 0.001), postoperative supportive treatment (radioiodine; HR: 5.829; 95% CI: 2.753, 12.341; *p* < 0.001), histologic subtype (WIFTC;HR: 28.232; 95% CI: 12.783, 62.354; *p* < 0.001), nodule size (≥40 mm; HR: 7.866; 95% CI: 3.633, 17.030; *p* < 0.001), calcification (HR: 3.256; 95% CI: 1.573, 6.739; *p* = 0.001), TSH (down and up, HR: 4.969; 95% CI: 2.543, 9.710; *p* < 0.001), and distant metastasis (M1, M2, HR: 660.682; 95% CI: 7.059, 54842.051; *p* = 0.004). After adjustment with multivariate Cox regression analysis of these 10 parameters, it was found that, with the exception of age, Hashimoto's thyroiditis, postoperative adjuvant therapy, calcification, and TSH, each was associated with an increased risk of cancer‐specific death, and each was a clinically independent prognostic factor: histologic subtype (adjusted HR: 7.440; 95% CI: 3.083, 17.954; *p* < 0.001), nodule size (adjusted HR: 8.622; 95% CI: 3.181, 23.369; *p* < 0.001), and distant metastasis (adjusted HR: 6.727; 95% CI: 2.488, 18.186; *p* < 0.001) (Table 3).

**TABLE 3:** Risk factors associated with cancer‐specific mortality in 153 patients with follicular thyroid cancer.

	Univariate	*p*‐value	Multivariate	*p‐*value
	HR (95% CI)		Adjusted HR (95% CI)	
Age (years)				
<55	Reference		Reference	
≥55	8.636 (3.535, 21.097)	<0.001	0.766 (0.450, 1.303)	0.325
Gender				
Female	Reference			
Male	1.048 (0.539, 2.039)	0.890		
Hashimoto's thyroiditis				
No	Reference		Reference	
Yes	2.372 (1.242, 4.530)	0.009	0.845 (0.565, 1.266)	0.414
Surgery method				
Unilateral lobectomy	Reference		Reference	
Total or near‐total unilateral lobectomy	0.339 (0.188,0.611)	<0.001	0.536 (0.271, 1.060)	0.073
Postoperative adjuvant therapy				
No	Reference		Reference	
Radioiodine therapy	5.829 (2.753, 12.341)	<0.001	1.298 (0.853, 1.974)	0.223
Histologic subtype				
Minimally invasive	Reference		Reference	
Widely invasive	28.232 (12.783, 62.354)	<0.001	7.440 (3.083, 17.954)	<0.001
Nodule size				
<40	Reference		Reference	
≥40	7.866 (3.633, 17.030)	<0.001	8.622 (3.181, 23.369)	<0.001
Calcification				
No	Reference		Reference	
Bulky/rim/punctate	3.256 (1.573, 6.739)	0.001	0.757 (0.498, 1.149)	0.191
Acoustic halo				
No	Reference			
Yes	0.188 (0.026, 1.383)	0.101		
Tumor aspect ratio				
<1	Reference			
>1	1.736 (0.529, 5.701)	0.363		
TSH				
Normal	Reference			
Down and up	4.969 (2.543, 9.710)	<0.001	0.760 (0.521, 1.110)	0.156
Group				
M0	Reference		Reference	
M1 and M2	660.682 (7.059, 54,842.051)	0.004	6.727 (2.488, 18.186)	<0.001

### 3.4 Cancer‐specific survival

**FIGURE 3:**
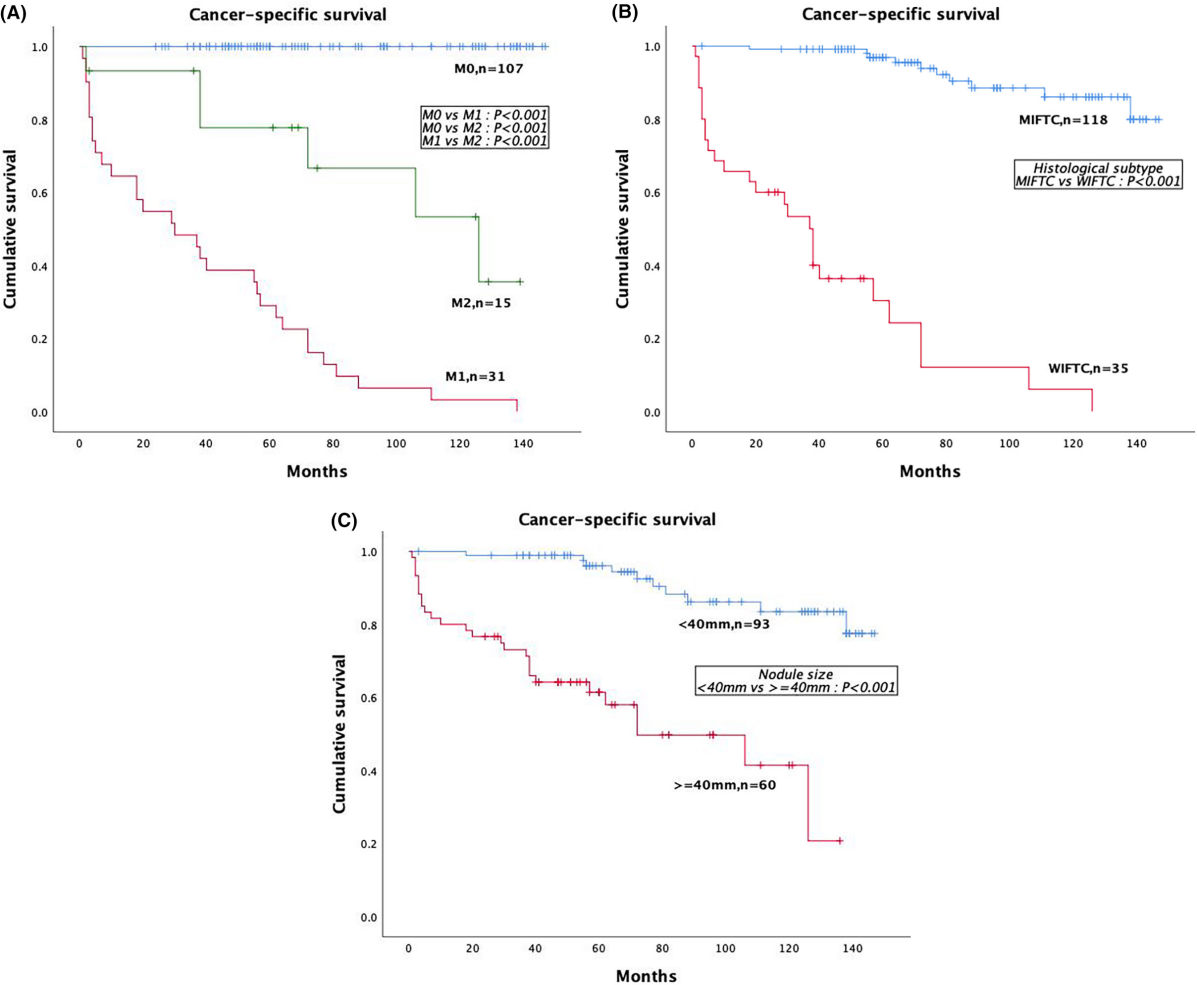
(A) Kaplan–Meier survival plots for patients with metastatic FTC at presentation (M1), developing metastasis during follow‐up (M2), and with no evidence of metastasis (M0). (B) Kaplan–Meier survival plots for FTC patients with MIFTC and with WIFTC. (C) Kaplan–Meier survival plots for FTC patients with the nodules are less than or egual to 40 mm in size.

The CSS was assessed using the Kaplan–Meier method and log‐rank test. It was found that the CSS was obviously distinct among the three groups. The M1 group has the poorest CSS. The 5‐year CSS in the M1 group was 25.8%, followed by the M2 (66.7%) and the M0 (100%) (all *p* < 0.001). The 10‐year CSS in the M1, M2, and M0 groups were at 3.2%, 35.6%, and 100%, respectively. The 5‐year and 10‐year survival rates of WIFTC patients were at 36.4% and 6.1%, respectively. This was in contrast to rates of 96.9% and 86.1%, respectively, for MIFTC (*p* < 0.001). The survival rates of FTC patients with node size > = 40 mm were lower compared with those node size <40 mm, with 5‐year survival rates of 58.0% for the former and 96.1% for the latter, respectively, and 10‐year survival rates of 20.7% for the former and 83.5% for the latter (*p* < 0.001), respectively. These results suggest that patients with FTC with widely invasive, distant metastases, or nodes size > = 40 mm have a worse prognosis (Table 4; Figure 3).

**TABLE 4:** Five‐year CSS rates, Ten‐year CSS rates of FTC patients with and without independent prognostic factors.

	Death	Survival	Five‐year CSS rate	Standard error	Ten‐year CSS rates	Standard error	*p*‐value
Histologic subtype							
Minimally invasive	10	108	96.9%	1.8%	86.1%	4.5%	
Widely invasive	27	8	36.4%	8.6%	6.1%	57.0%	<0.001
Nodule size							
<40	10	83	96.1%	2.2%	83.5%	5.2%	
≥40	27	33	58.0%	7.1%	20.7%	15.5%	<0.001
Group							
M0	0	107	100%	–	100%	–	
M1	31	0	25.8%	7.9%	3.2%	3.2%	
M2	6	9	66.7%	14.2%	35.6%	18.2%	<0.001

## 4 DISCUSSION

Our retrospective analysis revealed age disparities among the three groups of FTC patients. Some studies have suggested that older patients are more susceptible to distant metastasis.^21^ The association between HT and thyroid cancer is currently a subject of controversy, with the prevailing belief that HT is linked to an increased risk of developing thyroid cancer.^22^ In our study, the highest proportion of patients with HT and the least favorable prognosis in the M1 group seem to support this perspective.

Univariate Cox regression analysis showed that age, HT, surgery method, postoperative adjuvant therapy, histologic subtype, nodule size, calcification, TSH, and distant metastasis were correlated to prognosis significantly. Histologic subtype, nodule size, and distant metastasis were further determined as clinically independent prognostic indicators after adjusted multivariate Cox regression analysis for age, HT, surgery method, postoperative adjuvant therapy, histologic subtype, nodule size, calcification, TSH, and distant metastasis.

Due to the challenges associated with preoperative diagnosis, a step‐up surgical approach, involving restrictive primary resection (adeno lobectomy) followed by total thyroidectomy based on definitive histopathology, represents an alternative strategy to address this dilemma. A study employing multivariate analysis found that thyroidectomy was associated with lower survival free of distant metastases compared to complete resection.^3^, ^17^ Findings from a study of 324 MIFTC patients revealed that thyroid lobectomy (especially in patients aged ≥45 years) was linked to lower distant metastasis‐free survival in multivariate analyses compared to complete thyroidectomy.^16^ However, other studies contradict this conclusion, arguing that lateral thyroidectomy is sufficient for MIFTC patients, while WIFTC necessitates total thyroidectomy and postoperative radioiodine therapy to enhance survival rates and control local recurrence.^19^ Another study suggests that the prognoses for total thyroidectomy and unilateral lobectomy are similar regardless of FTC size, as long as there are no distant metastases at the time of diagnosis, and unilateral lobectomy is unnecessary unless the patient experiences recurrence.^18^ The choice of surgical extent for FTC patients remains a contentious issue in existing studies, and no significant difference in prognosis between pneumonectomy and unilateral lobectomy was determined in this study. Further research is needed to establish individualized surgical approaches for FTC patients.

**FIGURE 4:**
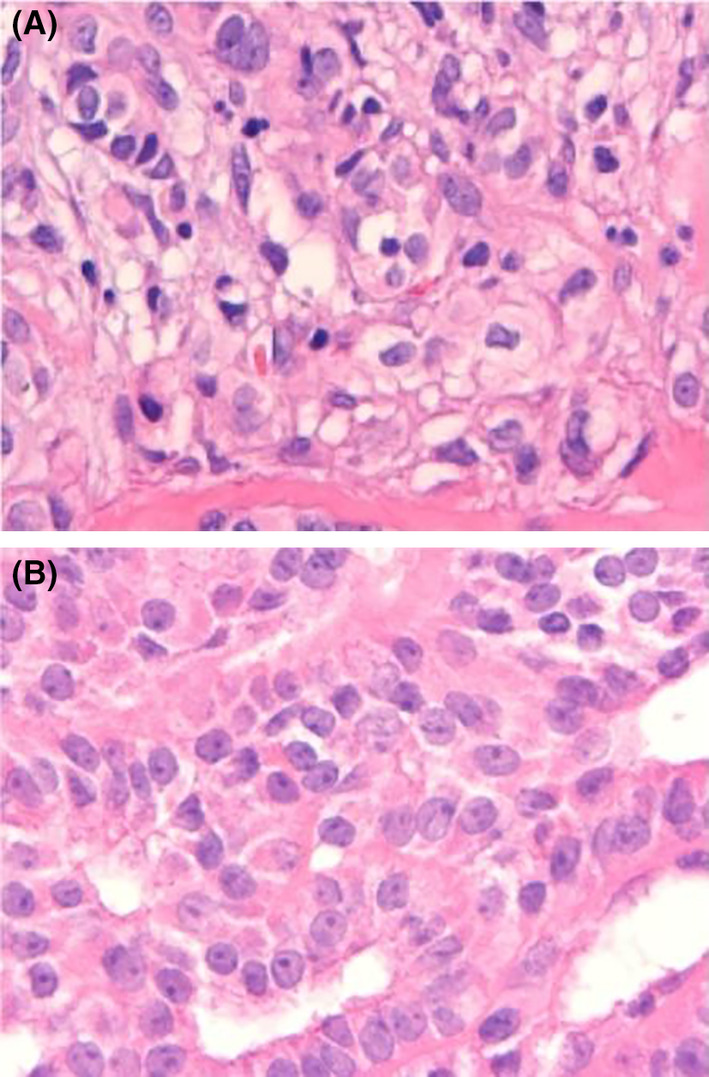
(A) Histologic pathology image of MIFTC. (B) Histologic pathology images WIFTC.

Hürthle cell carcinoma is an inappropriately used term that has been replaced in the fifth edition of the WHO Classification of Thyroid Tumors by thyroid neoplastic cell carcinoma, a separate entity distinct from FTC.^23^ The WHO continues to classify FTC into three histologic subtypes: minimally invasive, angio invasive, and widely invasive.^23^ It also mentions capsular invasion and/or vascular infiltration as features of FTC, without the typical nuclear features of PTC.^24^ Due to the vascular invasiveness of FTC, it is more likely to metastasize to distant organs and not only to regional lymph nodes.^25^ Some studies have indicated that WIFTC is a poorer prognosis than MIFTC, which is consistent with our study.^26^, ^27^ Other research has found no significant difference in disease‐free survival between patients with MIFTC who underwent thyroidectomy alone and those who received thyroidectomy and radioiodine therapy after thyroidectomy.^28^ This is another evidence of the better prognosis of MIFTC. Without classifying angio invasive FTC separately (Figure 4), the highest WIFTC rate in this study was seen in the M1 group, while the highest MIFTC rate was observed in the M0 group. In alignment with other studies, we also conclude that WIFTC has a worse prognosis. MIFTC has a good prognosis, while AIFTC and WIFTC exhibit higher risks of metastasis and recurrence.

Moreover, we have found good prognosis in the patients who had FTC without remote metastases, with a 10‐year CSS at 100% in our study. In contrast, patients with distant metastasis found either at the time of diagnosis or at follow up had a poor prognosis. Several studies have shown that there is no apparent difference in the short time period between the survival of patients with FTC who have metastases at diagnosis and those who are found to have metastases after treatment.^2^ On the contrary, our research and that by Wu et al.^29^ found that the patients with distant metastases at diagnosis had a lower survival rate. Individuals who present with metastatic FTC should therefore be treated aggressively with thyroidectomy, radioiodine ablation, and resection of the metastasis where possible.^2^ Some studies found that age, lesion size, histology, vascular invasiveness, extraglandular invasiveness, and metastasis were found to be risk factors for increased tumor‐specific mortality in patients with FTC.^21^, ^30^, ^31^ Several independent risk factors for FTC are reported in the literature, including increased nodule size (>40 mm), which were consistent with our findings.^3^ The prognosis of small lesions of WIFTC is worse than that nodule size >40 mm of MIFTC, which shows that WIFTC has a greater aggressiveness.^32^ Our study only found that pathologic categorization, nodule size, and distant metastasis were independent risk factors for CSM. Several studies have found that mutations in the RAS gene can occur in diagnosed thyroid tumors, both benign, and malignant, and are most common in FTC, but *RAS* seems not to be a prognostic factor for thyroid disease.^33^ Low levels of DUSP5 in FTC are involved in proliferation, migration, and invasion, which further leads to poorer prognosis. Therefore, DUSP5 might serve as a novel therapeutic target for FTC,^34^ it remains to be further researchers.

We have some limitations similar to other studies. First, the large time frame of the sample included in our study, for the surgical approach and postoperative adjuvant treatment, despite the guidelines guiding standardized treatment, the current level of technology must have improved compared to the previous ones, and the surgeons have their own treatment options without violating the treatment principles, which would give rise to a situation where patients with similar conditions have different prognoses. There was no significant difference in prognosis between total and unilateral lobectomy in our study, unlike the results of other studies, which may be closely related to the significant improvement in the level of medical technology in recent years. Furthermore, retrospective studies may be influenced by patient selection criteria, and patients initially classified as low risk and consequently receiving less aggressive treatment may potentially introduce interference into the results. We plan to complement our study with additional multicenter research, incorporating more refined subgroups, to mitigate such interference. Second, the absence of distant metastases in all included samples does not necessarily mean that distant metastases did not really occur; it is possible that small distant metastases were not detected or that patients avoided some imaging examinations without corresponding symptoms or for other reasons, such that distant metastases were not diagnosed, which has a direct impact on the results. Third, despite statistical adjustment for multiple factors, unknown confounding factors, such as patient comorbidities, relevant molecular markers (e.g., BRAF, RAS, TERT, etc.), can have an impact on the results. Therefore, there will be some deviation from the actual situation. Compared to other relevant studies, this study included data from three hospitals with a relatively large sample size, which can offset some of the bias, and this is the main advantage of this study.

## 5 CONCLUSION

The definitive diagnosis of follicular tumors relies on postoperative histologic assessment. Until improved diagnostic tools become available, our ability to identify high‐risk populations remains limited to stratifying patients based on clinical risk factors. In this study, we identified FTC characteristics associated with cumulative mortality risk, including histologic subtype, nodule size, and distant metastasis. Patients with FTC lacking distant metastases exhibited a more favorable prognosis, whereas those with distant metastases, whether present at initial diagnosis or during follow‐up, faced a poorer survival outlook. FTC patients with distant metastases, nodules measuring ≥4 cm, and extensive invasiveness therefore necessitate more aggressive treatment. Increased surveillance would enable clinicians to detect individuals with an unfavorable FTC prognosis at an earlier stage. Consequently, we plan to initiate a prospective multicenter study encompassing comprehensive FTC management, including the determination of surgical scope and postoperative adjuvant therapy. This approach aims to offer more personalized.

## AUTHOR CONTRIBUTIONS

**Jiafei Shen:** Conceptualization (equal); data curation (lead); formal analysis (equal); investigation (lead); writing – original draft (lead); writing – review and editing (equal). **Meiying Yan:** Conceptualization (equal); formal analysis (lead); writing – review and editing (equal). **Long Chen:** Investigation (equal). **Di Ou:** Supervision (equal). **Jincao Yao:** Funding acquisition (equal); supervision (equal). **Na Feng:** Funding acquisition (equal). **Xueqin Zhou:** Writing – review and editing (equal). **Zhikai Lei:** Supervision (equal). **Dong Xu:** Funding acquisition (lead).

## FUNDING INFORMATION

This study was supported in part by the National Natural Science Foundation of China (Nos. 82071946), the Zhejiang Provincial Natural Science Foundation of China (No. LZY21F030001 and LSD19H180001), the Research Program of Zhejiang Provincial Department of Health (Nos. 2021KY099 and 2022KY110), and the Project of Zhejiang Medical and Health Science and Technology Plan (No. 2023KY066).

## CONFLICT OF INTEREST STATEMENT

All authors declare no conflicts of interest concerning this work.

## Data Availability

N/A
